# Hollow-Fibre-Supported Dispersive Liquid-Liquid Microextraction for Determination of Atrazine and Triclosan in Aqueous Samples

**DOI:** 10.1155/2017/1451476

**Published:** 2017-10-11

**Authors:** Thabiso Letseka, Mosotho J. George

**Affiliations:** Department of Chemistry and Chemical Technology, National University of Lesotho, P.O. Roma 180, Maseru, Lesotho

## Abstract

We report the application of the dispersive liquid-liquid microextraction coupled to hollow-fibre membrane-assisted liquid-phase microextraction and its application for extraction of atrazine and triclosan. Under optimum conditions, namely, 25 *μ*L of a 1 : 4 chlorobenzene : ethyl acetate mixture dispersed in 1 mL of aqueous sample, 10% (m/v) NaCl, a magnetic stirrer speed at 600 rpm, and 10 minutes' extraction time with toluene-filled fibre as the acceptor phase, the method demonstrates sufficient figures of merit. These include linearity (*R*^2^ ≥ 0.9975), intravial precision (%RSD ≤ 7.6), enrichment factors (127 and 142), limits of detection (0.0081 and 0.0169 *µ*g/mL), and recovery from river water and sewerage (96–101%). The relatively high detection limits are attributed to the flame ionization detector which is less preferred than a mass spectrometer in trace analyses. This is the first report of a homogenous mixture of the dispersed organic solvent in aqueous solutions and its employment in extraction of organic compounds from aqueous solutions. It therefore adds yet another candidate in the pool of miniaturised solvent microextraction techniques.

## 1. Introduction

The use and access to clean and safe water is a worldwide concern as to prevent any water-borne illnesses, and this has led to implementation of water quality regulations thus the need for improvements in water treatment technologies. This is driven by the emerging contaminants in the environment that are present as a result of anthropogenic activities which include agriculture, household processes, and industrial processes. These compounds inadvertently find their way into environmental water through production, use, and final disposal of materials containing these compounds [1]. They are generally discharged off in low concentrations into the environment; however as a result of their nonbiodegradability they tend to bioaccumulate in the environment which they are deposited in [2, 3]. It is essential therefore to monitor the presence and concentrations of these compounds in the environment in order to reduce their effects on biodiversity.

Since these compounds are present in the environment at relatively low concentrations and have to be isolated from complex matrices, this necessitates sample preparation for removal of interferences and preconcentration of target analytes [4]. Traditionally, liquid-liquid extraction was the most widely used sample preparation technique but had a number of drawbacks which include chiefly utilization of large volumes of solvents and time consumption [5]. In an effort to overcome the said drawbacks, a number of new miniaturised techniques have been developed and are continuously receiving attention from analytical chemists. These techniques offer improvements such as simplicity of operation, rapidity, low cost, less hazards, and high enrichment factors [6].

There are principally three modes of miniaturised liquid-liquid extraction, sometimes called liquid-phase microextraction (LPME) or solvent microextraction, namely, single drop microextraction (SDME) [7, 8], hollow-fibre liquid-phase microextraction (HF-LPME) [9], and dispersive liquid-liquid-phase microextraction (DLLME) [10]. Lastly, DLLME entails the addition of appropriate mixture of extracting and dispersing solvents to an aqueous sample containing analytes of interest; then formation of a cloudy solution occurs. When the cloudy solution is centrifuged, the extracted solvent will sediment at the bottom of the tube and is then withdrawn with a microsyringe for instrumental analysis [11]. This method has received a wide acceptance for use in analysis of environmental pollutants due to its unmatched rapid extraction rates. However the need for precipitation of the organic solvent or aqueous to separate the two phases still introduces an extra step in the analysis time and as such this presents another input cost in the form of a centrifuge.

Hollow-fibre liquid-phase microextraction on the other hand involves the use of a hollow-fibre membrane to support the organic solvent. It is fast, efficient, sensitive, and easy to operate and high enrichment process. The high efficiency of HF-LPME is related to the high mass transfer rates of the process [12]. The type of solvent to be filled and immobilized in the pores of the hollow fibre is a critical factor to consider as the solvent must be compatible with the propylene fibre so as to easily fill the pores, and it should also be immiscible with the water sample and provide high affinity of the target analytes into it with low volatility to avoid evaporation during the extraction but have sufficient vapour pressure to be evaporated in the GC injection port [13–15]. Usually the extraction kinetics and enhanced mass transfer of target analytes from the donor phase to the acceptor phase are achieved through stirring of the sample [14].

Efforts to combine some of these techniques with potential complementarity have demonstrated a promise in this regard. For example, a combination of solid phase microextraction with dispersive liquid-liquid microextraction [16] and the combination of electromembrane extraction with DLLME [17] have been reported. Recently an attempt was reported using a modified DLLME to drive the analytes into the headspace followed by solid phase microextraction [18]. The potential of yet another combination of two different techniques: DLLME with HF-LPME by introducing the organic solvent filled fibre into the cloudy DLLME solution as a way of by-passing the extra step of phase separation has been reported [19]. Despite the reported challenge of the precipitation of the more dense organic solvents, this method was applied in the determination of triclosan in sewage water with considerable success [20]. However, there was still a challenge of phase separation between the two solvents—aqueous solution and the organic solvent used in the DLLME.

As rigorous attempts were made to improve this situation—phase separation after the dispersion of the organic solvents in the aqueous solution—we herein report the optimised coupled HF-LPME with DLLME using a binary organic solvent without any phase separation, thus achieving the benefit of the two techniques as reported before as well as a more improved precision which was a challenge with the previously reported method which had some precipitation of the organic solvent, thus posing a challenge of avoiding the sedimented droplets or suspended organic solvents. Atrazine and triclosan were used as model organic pollutants in this work as a follow-up to the previous exercise.

## 2. Experimental

### 2.1. Materials and Reagents Used

Atrazine (1-chloro-3-ethylamino-5-isopropylamino-2,4,6-triazine, CAS 1912-24-9) was purchased from Chem Service, Pennsylvania, USA, and triclosan [5-chloro-2-(2,4-dichlorophenoxy)phenol, CAS 3380-34-5], analytical grade diphenylamine, chlorobenzene, and ethyl acetate were obtained from Sigma Aldrich (Johannesburg, South Africa) while the HPLC grade solvents—methanol, ethanol, toluene, and chloroform—were obtained from Riedel-de Haën (Seelze, Germany); analytical grade NaCl was obtained from ACE (Johannesburg South Africa). The distilled water was prepared in house. The Accruel Q3/2 PP polypropylene hollow-fibre membrane with the dimensions of 600 *μ*m (internal diameter) × 200 *μ*m (wall thickness) × 0.2 *μ*m pore size was obtained from Membrana GmbH (Wuppertal Germany) and cut into 1 cm strips using a measuring ruler and a pair of scissors. Spinbar® magnetic stirring fleas with 5 mm × 2 mm dimensions were obtained from Sigma Aldrich (Steinheim, Germany).

Stock standard solutions were prepared by dissolving 10 mg of atrazine and triclosan standards in 1 mL ethanol, and this solution was further diluted serially to achieve lower concentrations using ethanol. Aqueous working solutions of 5 *µ*g/mL were prepared by dilution of determined volumes of the stock standard solutions with distilled water. All the solutions were stored in the refrigerator at temperature below 5°C when not in use.

### 2.2. Instrumentation

The analysis was carried out using a Varian 3800 Gas Chromatograph (California, USA) equipped with a flame ionization detector and a 30 m × 1 *μ*m × 0.53 mm SGE-BP5 (5% phenyl-95% dimethyl-polysiloxane) column (Texas, USA). Nitrogen gas (5.0 Grade) was used as a carrier gas and maintained at 5 mL/min while hydrogen and air were used for the detector. The injector and detector temperature were set at 250°C and 200°C, respectively. The column was held at 100°C for 2 min, then ramped at 20°C/min to 300°C, and held for 3 min to achieve a total run time of 15 minutes.

### 2.3. Extraction Procedure

The extraction procedure was followed as described elsewhere [19]. Firstly, 1 mL aliquots of the 5 *µ*g/mL working solution were pipetted into a 2-mL sample vial with a screw-cap to which a 25 *µ*L mixture of 1 : 4 chlorobenzene : ethyl acetate was then pipetted; 15 mg NaCl was weighted and then added to the solution. The vial contents were shaken vigorously to achieve homogeneity and dispersion of the extraction phase into fine droplets. Thereafter a Spinbar magnetic stirring fleas was also introduced in the solution and the vial was placed on the magnetic stirrer hotplate. Thereafter a 1 cm long hollow-fibre membrane filled with toluene acceptor solvent (prespiked with the diphenylamine internal standard) fitted at the tip of the Hamilton® syringe was introduced carefully into the solution and the vial was tightly capped. The magnetic stirrer hotplate (Stuart® Scientific, Staffordshire, UK) was set to 600 rpm to keep the dispersed fine droplets of the extraction phase suspended in the aqueous phase for a 10-minute extraction period. After the extraction time had elapsed, 3 *μ*L was withdrawn and injected into the gas chromatograph for analysis.

### 2.4. Sampling

The real samples were collected in triplicate in 50 mL Schott® bottles and stored below 5°C until further use. The river sample was obtained from Liphiring river 3-4 km northwest of the NUL Roma campus few meters upstream of the road bridge to avoid potential pollution from the traffic, while the sewage water was obtained from the old sewerage treatment ponds north of the NUL Roma campus that still collects an overflow from the main pipes carrying the sewerage to the newly built treatment ponds about 3 km further north of the campus. Prior to the extractions, these samples were allowed to warm to room temperature.

For the recovery experiments, 1-mL portions of river and sewage water samples were spiked with the stock solution of the mixture of the two analytes to obtain concentrations of 25 *µ*g/mL. Thereafter, a similar extraction as outlined in the extraction procedure was carried out under the optimum conditions with the responses compared against the HPLC grade water spiked at the corresponding concentrations.

## 3. Results and Discussions

### 3.1. Investigation of Extraction Conditions

#### 3.1.1. Selection of the Extracting Solvent Mixture without Phase Separation

Selection of the extracting solvent is often based on the solubility of the target compounds and immiscibility in water so as to cause a formation of fine droplets that can be dispersed in the sample which will lead to an increase in surface area between the extracting solvent and the aqueous sample as to make the extraction independent of time [5]. The task in this exercise was to determine a mixture of two solvents that would result in the sustenance of the organic solvent mixture in the aqueous solution to ensure homogenous mixture and to prevent phase separation and its inherent limitations such as a decrease in the preconcentration and precision [16]. A mixture of two solvents was then made consisting of two solvents one being more dense than water (chlorobenzene) with the other being less dense such that the final density of the mixture would closely match that of water so as to minimize any sedimenting or floating solvent. This mixture was made by varying the proportions of ethyl acetate and chlorobenzene such that when the mixture is dispersed in the aqueous phase a cloudy solution will be formed with less or no separation. A 1 : 4 mixture of ethyl acetate : chlorobenzene was found to be the optimum mixture ensuring the homogenous mixture of the organic solvent and the aqueous solution, and consequently it was selected as an ideal solvent mixture to be used in the further experiments.

#### 3.1.2. Effect of Varying Solvent Mixture Volume

To determine the effect of extracting mixture volume on the extraction efficiency, the working solutions were spiked with varying volumes of the 1 : 4 chlorobenzene : ethyl acetate mixture in the range of 0–50 *µ*L under 10 minutes' extraction time. The relative extraction was calculated as a ratio of the peak areas of the analytes to that of the internal standard.

**Figure 1:**
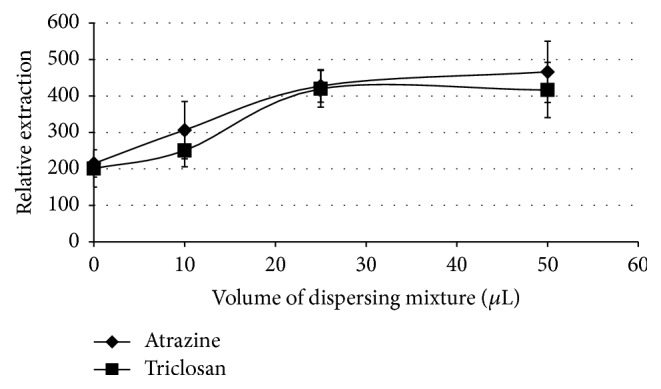
The effect of varying the volume of the extracting organic solvent on the extraction. Conditions: 1 mL of 5 *µ*g/mL triclosan and atrazine aqueous sample volume, varying volumes of a 1 : 4 (v/v) chlorobenzene : ethyl acetate mixture with the extraction time of 20 minutes and with toluene as the acceptor solvent. The relative extraction was calculated as a ratio of the peak areas of the analyte to that of the internal standard multiplied by 100.

As can be seen from Figure 1, extraction increases with increasing volume of the extracting mixture and plateaus between 25 and 50 *µ*L. The slight drop at 50 *µ*L could be due to the dilution of the analytes in the predispersed solvents thus leaving considerable amount in the solution, as well as the fact that the acceptor solvent in the fibre cannot dissolve the whole predispersed volume as argued previously [19]. Consequently, 25 *µ*L of the organic solvent mixture (chlorobenzene : ethyl acetate) was selected to be the optimum volume for the extracting solvent mixture and thus used for further experiments.

#### 3.1.3. Effect of Acceptor Phase

The type of solvent to be filled and immobilized in the pores of the hollow fibre is a critical factor to consider as the solvent must be compatible with the propylene fibre so as to easily fill the pores, and it should also be immiscible with the aqueous sample and provide high affinity of the target analytes into it with low volatility to avoid evaporation during the extraction but have enough vapour pressure to be evaporated in the GC injection port [13–15]. Four different organic solvents, namely, chlorobenzene, toluene, ethyl acetate, and dichlorobenzene, and the mixture of ethyl acetate and chlorobenzene were selected to fill and impregnate the pores of the hollow fibre.

**Figure 2:**
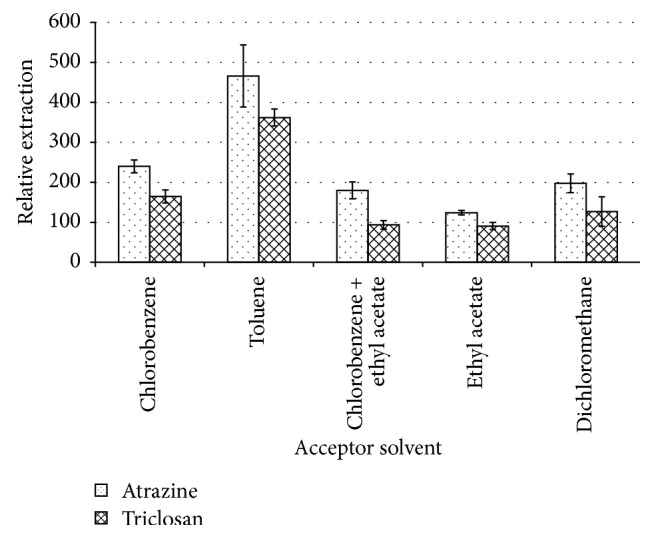
Effect of varying the acceptor solvent on the extraction efficiency. Conditions: volume, 1 mL of 5 *µ*g/mL atrazine and triclosan aqueous sample; 25 *µ*L of a 1 : 4 (v/v) chlorobenzene : ethyl acetate mixture with the extraction time of 20 minutes.

As can be seen in Figure 2, the best extraction was obtained using toluene as the acceptor phase since toluene has almost all the qualities mentioned above that a good acceptor solvent should possess and the presence of a benzene ring in its structure enables faster transfer of analytes into it based on the like dissolves like principle [15]. These results are consistent with those reported elsewhere [20]. Toluene was therefore selected as the ideal acceptor solvent for further experiments.

#### 3.1.4. Effect of Ionic Strength

Typically ionic strength increases extraction efficiency as a result of the commonly known salting-out effect—decreasing the solubility of analytes in the aqueous phase thus enhancing partition into the organic phase [12, 14]. However this phenomenon does not hold to an infinite increase in salt concentration, as the concentration increases to some point the extraction efficiency decreases which lowers the enrichment factor, and this may be due to an increase in viscosity of the solution which may lower the mass transfer rate and/or the change in physical properties of the Nernst diffusion layer [16, 21]. Besides this, ionic strength has been reported to increase the stability of the organic droplets in the drop-based microextraction techniques [22, 23]. Consequently, the effect of addition of the sodium chloride to the aqueous samples prior to extraction was investigated.

**Figure 3:**
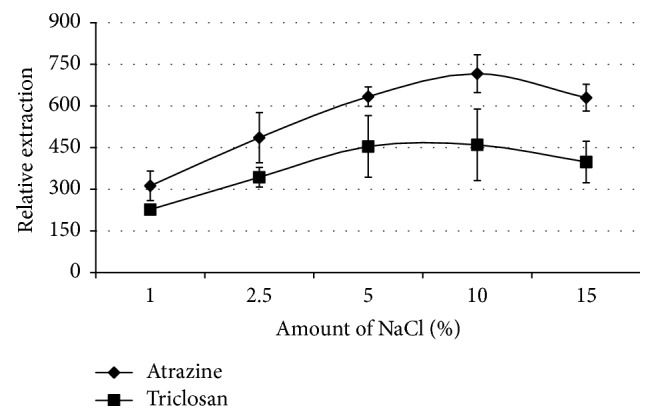
The effect of adding sodium chloride on the extraction. Conditions: volume, 1 mL of 5 *µ*g/mL triclosan and atrazine aqueous sample; 1 : 4 (v/v) chlorobenzene : ethyl acetate mixture with the extraction time of 20 minutes and with toluene as the acceptor solvent.

It is evident from Figure 3 that the extraction of the two analytes increased with increasing salt addition and peaked at 10% salt concentration and then dropped as concentration of the salt was further increased to 15%. This drop is argued on the basis of salting out of the organic solvent from the aqueous solution thus rendering the solvent unavailable to the analytes and the extreme being the floating of the organic solvent (for less dense solvents than water) thus taking the analytes to the surface and being not available to the fibre that is introduced into the solution [24]. This therefore offsets the salting-out effect and thus leads to a drop in extraction efficiency.

#### 3.1.5. Effect of pH

The value of ion activity, commonly known as pH, is very important especially for compounds that possess either acidic or basic functional groups. Owing to the structures of both compounds, the presence of an acidic OH group on triclosan, and two basic > NH units on atrazine, it would therefore be interesting to explore the effect of pH on these potentially conflicting functional groups. The effect of pH was therefore explored with the aqueous phase pH of 2 and 4 adjusted by HNO_3_ and the aqueous pH of 10 and 12 adjusted by NaOH.

**Figure 4:**
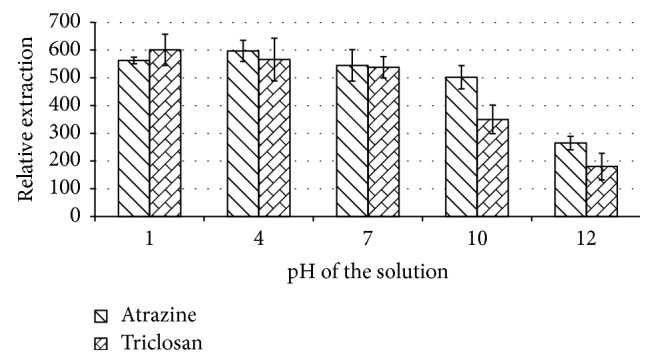
The effect of pH of the aqueous solution on extraction. Conditions: volume, 1 mL of 5 *µ*g/mL atrazine and triclosan aqueous sample to which 15 mg NaCl was added (15% w/v); 25 *µ*L of a 1 : 4 (v/v) chlorobenzene : ethyl acetate mixture with the extraction time of 20 minutes and with toluene as the acceptor solvent.

As can be seen from Figure 4, the analytes extract is better in acidic media. This is due to the potential acidic nature of triclosan. However, the same trend was observed with atrazine, which was inexplicable given that atrazine was expected to be basic due to the >NH groups. This anomaly could be possibly attributable to the weak basicity of the secondary amino groups compared to the primary amine (-NH_2_). Given the corrosive effect of strong acid media on the metallic syringe, the pH of the subsequent solutions was left at neutral since the increase with lower pH was not considerable (less than 10% on average) for both analytes between the pH values of 7 and 2.

#### 3.1.6. Effect of Stirring Rate

One of the major advantages of HF-LPME is that since the accepter phase is supported in the lumen of the fibre, this can tolerate high stirring speeds without any losses of the acceptor phase [25].

**Figure 5:**
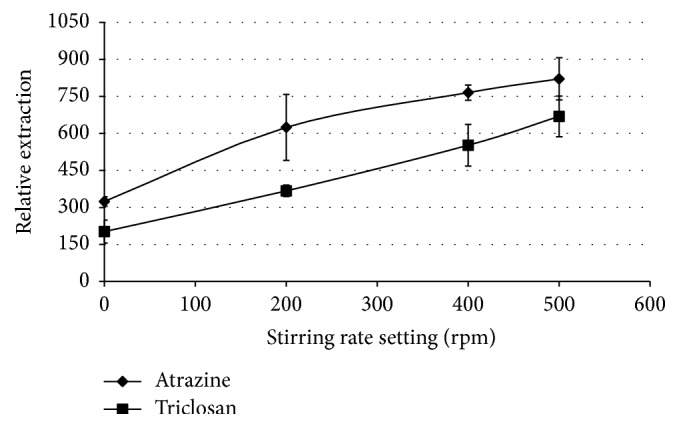
Effect of stirring the aqueous solution on the extraction of the analytes. Conditions: volume, 1 mL of 5 *µ*g/mL atrazine and triclosan aqueous sample to which 15 mg NaCl was added (15% w/v); 25 *µ*L of a 1 : 4 (v/v) chlorobenzene : ethyl acetate mixture with the extraction time of 20 minutes and with toluene as the acceptor solvent.

Figure 5 shows that when increasing the stirring speeds the extraction efficiency increases and this can further be explained by the fact that stirring the solution increases the contact between the preextracted analytes in the extracting solvent and the fibre [26], as well as preventing the formation of a Nernst diffusion layer which reduces the concentration gradient at the interface of the fibre and the bulk aqueous solution. Despite the observed increased extraction, some vortex was developed and irritated the fibre leading to some fibre dislodgement at the stirring speed settings greater than 600 rpm, and thus the stirring speed of 600 rpm was set as the optimum stirring speed and used in subsequent experiments.

#### 3.1.7. Effect of Extraction Time

The two techniques coupled in this study demonstrate contrasting time dependence with HF-LPME requiring longer time than DLLME which takes only seconds to reach equilibrium. This then called for determination of the optimum extraction time for the method and this was investigated in the time range of 5–20 minutes.

**Figure 6:**
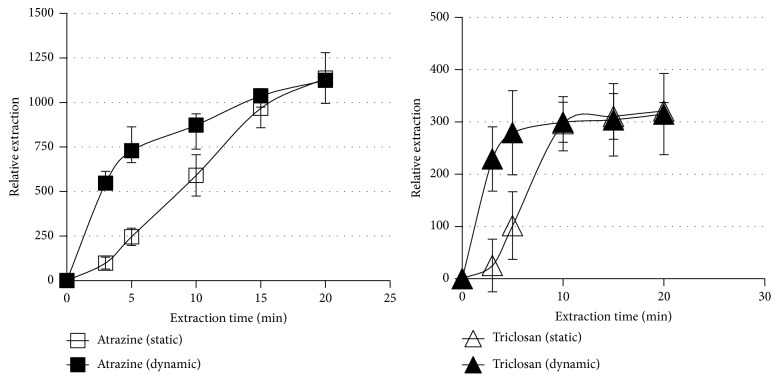
The extraction-time profile for the two analytes with and without stirring. The extraction-time profile of triclosan under the optimised extraction conditions: volume, 1 mL of 5 *µ*g/mL atrazine and triclosan aqueous sample to which 15 mg NaCl was added (15% w/v); 25 *µ*L of a 1 : 4 (v/v) chlorobenzene: ethyl acetate mixture; toluene as the acceptor solvent; stirring rate 600 rpm and a pH of 7.

As can be seen from Figure 6, especially in the case of triclosan, the extraction reached saturation in about 5 minutes with stirring which is less than half the time (10 min) taken without stirring. However, less effect was observed with atrazine which demonstrated less saturation although the extraction shows two different rates with a more rapid uptake less than 5 minutes followed by a less rapid and gradual increase between 5 and 20 minutes where the extraction values with and without stirring intersect with one another. The rapid uptake of triclosan could be attributed to its less solubility in water (0.001 g in 100 mL at 25°C) [27] than atrazine (30 mg/litre at 20°C) [28]. The ideal extraction time was therefore set at 10 minutes for the subsequent extractions.

### 3.2. Method Validation

The optimum conditions can be summarized as follows: spiking of sample solutions with 25 *μ*L of a 1 : 4 mixture of chlorobenzene : ethyl acetate, use of toluene as the acceptor phase, 10% (m/v) NaCl, a magnetic stirrer speed at 600 rpm, and 10 minutes' extraction time. Following optimisation, the method was applied to the river and sewage water samples spiked at different concentration levels (0.5–50 *µ*g/mL) to determine linearity, the precision, and other analytical parameters such as limits of detection and coefficient of determination (see Table 1). The enrichment factors were determined by directly injecting an organic solution of the analytes and the extract of the aqueous solution at the corresponding concentration.

The observed coefficients of determination (0.9975 atrazine and 0.9977 triclosan) demonstrate that this technique is sufficiently linear in the range explored. The method demonstrated sufficiently low limits of detection (0.0081 atrazine and 0.0169 triclosan) given that the detection method used was a flame ionization (FID) that is less robust than a mass spectrometer. Previous reports demonstrated that the latter is about three orders of magnitude more sensitive than the FID [19]. This therefore suggests that the limits of detection would be well in the parts per trillion (ng/L) range. Interestingly, triclosan demonstrates a higher limit of detection than atrazine possibly due to more chlorine atoms (3) than atrazine (1) which are known to have a quenching effect on the FID hence electron capture detector being more preferable for organochlorinated compounds. Despite this, the method demonstrated sufficient intravial precision with the %RSD ≤ 7.6 (*n* = 5).

The application of the method in the determination of both analytes in the river water and sewage could not detect these analytes. It must be mentioned that since the previous efforts using a better instrument, GC-MS, could not detect these compounds in the previous studies [19, 20] it was not expected that these compounds would be detectable. The approach was more to study the matrix effect from the real water samples on the recovery of these analytes, wherein the method was thus satisfactory in that the recovery was in the acceptable range (96–101%) indicating that the method does not suffer much from the matrix effect resulting from the two samples.

### 3.3. Comparison of the Developed Method with Existing Methods for Determination of the Selected Analytes

The comparison of some of the figures of merit obtained in this method with those reported in literature was made as summarized in Table 2. These included limits of detection, precision, and enrichment factors. Other parameters such as linearity, recovery, and accuracy were not included for clarity of the picture mostly while recovery was left out since the matrices for which the recoveries were obtained were different ranging from river water to soil to food samples. As can be seen from Table 2, the currently reported method, despite using a somewhat poor detector, namely, an FID, demonstrated sufficiently low LOD that is about 10x obtained with a classical DLLME coupled with a GC-MS as reported by Guo et al. [29]. This is remarkable given that FID is about 1000x less sensitive than an MS detector. Another issue concerning the LODs is that the reported LOD in this method is that achieved through the calibration approach which has been demonstrated to depend considerably on the slope and the error in the calibration and is usually higher than those reported from the S/N ratio approach which is the most commonly reported approach in cited literature [30].

Regarding the enrichment factors, only a few reports were accessed where the enrichment factors were stated for the target analytes. However, for those reports where these were stated, the current method demonstrates far superior enrichment factors which makes this approach a good candidate for preconcentration of analytes for trace analysis. The reported precision, whether dealt with as repeatability or reproducibility, matches those reported in literature; importantly these fall within the acceptable range adopted from trace analysis.

## 4. Conclusion

This report presents the development of the homogenous organic solvent with aqueous solution and its application in solvent microextraction, specifically the recently reported coupled HF-LPME with DLLME. The recovery values indicate the method does not suffer considerably from the matric effects from the river and sewerage water samples.

## Figures and Tables

**Table 1:** Some analytical data obtained from the regression analysis.

Analytical data	Atrazine	Triclosan
Coefficient of determination, *R*^2^	0.9975	0.9977
Estimated LOD (*μ*g mL^−1^)^*∗*^	0.0081	0.0169
Enrichment factor, EF^#^	127	142
Amount in river water sample^$^	n.d. (98)	n.d. (101)
Amount in sewage water	n.d. (97)	n.d. (96)
Reproducibility (%RSD, *n* = 5)	6.9	7.6

^*∗*^Limit of detection, LOD, calculated from the equation LOD = (3 × error  of  intercept)/slope; ^#^EF = *C*_org_/*C*_aq_. ^$^The values in parenthesis demonstrate the percentage recovery of the spiked samples.

**Table 2:** Comparison of some analytical parameters for this method with those reported in literature for the same analytes.

	Method	LOD (ng mL^−1^)	Precision (%RSD)	EF^*∗*^	Ref
Atrazine	DLLME-GC-MS	0.1	6.8		[29]
	QuECHERS-GC-MS	0.01	4.8		[31]
	VALLME-SFO-LC-MS/MS^#^	0.026	9.1	49	[32]
	DLLME-SFO-LC-MS/MS	0.029	11.1	37	[32]
	DLLME-LPME-GC-MS	0.063	10.7	62	[19]
	*DLLME-LPME-GC-FID*	*0.81*	*6.9*	*127*	*This work*

Triclosan	SPE-HPLC	0.134			[33]
	VALLME-SFO-LC-MS/MS	0.083	7.7	51	[32]
	DLLME-SFO-LC-MS/MS	0.129	7.2	45	[32]
	MEPS- LVI-GC-MS^*δ*^	0.003	2.2		[34]
	DLLME-SFO-LC-UV (LC-MS)^$^	0.10 (0.002)	4.4 (4.6)		[35]
	*DLLME-LPME-GC-FID*	*1.69*	*7.6*	*142*	*This work*

^*∗*^Enrichment factor calculated as a ratio of the relative response after the extraction to that of the original solution; ^#^VALLME-SFO stands for vortex assisted liquid-liquid microextraction based on solidification of floating organic solvent; ^*δ*^MEPS stands for microextraction by packed sorbents. ^$^The values in parentheses denote the analytical technique used after the sample preparation method.
